# Fibrin Monomer in Thrombosis and Haemostasis: A Clinical Biomarker and Beyond

**DOI:** 10.3390/ijms262411822

**Published:** 2025-12-07

**Authors:** Konstantin Guria, Ivan Melnikov, Valentina Shtelmakh, Yuliya Avtaeva, Sergey Okhota, Olga Saburova, Sergey Kozlov, Zufar Gabbasov

**Affiliations:** 1 National Medical Research Centre of Cardiology Named After Academician E.I. Chazov, Ministry of Health of the Russian Federation, 15A Akademika Chazova Street, 121552 Moscow, Russia; 2 State Research Center of the Russian Federation, Institute of Biomedical Problems of Russian Academy of Sciences, 76A Khoroshevskoye Shosse, 123007 Moscow, Russia

**Keywords:** fibrin, fibrin monomer, soluble fibrin monomer complexes, biomarker, blood coagulation, thrombosis, haemostasis regulation

## Abstract

Fibrin monomer (FM) is a transient intermediate of blood coagulation that functions as both an active regulator of haemostasis and a sensitive biomarker for prothrombotic states. Clinically, FM is measured indirectly as its derivative, soluble fibrin monomer complexes (SFMC), which is also often referred to as FM throughout the clinical literature. FM participates in a complex regulatory network modulating thrombin generation and fibrinolysis, interacting with platelet receptors, including integrin αIIbβ3 and GPVI, and engaging GPIb-vWF interactions. This comprehensive review examines FM’s molecular mechanisms in haemostatic regulation and evaluates clinical evidence for FM as a biomarker. Particular focus is placed on FM’s utility for risk stratification across thrombotic conditions, including disseminated intravascular coagulation, venous thromboembolism, ischemic stroke, myocardial infarction, and COVID-19-associated coagulopathy. Current challenges, including assay standardization and universal cut-off values, are discussed. By synthesizing mechanistic insights with clinical data, this integrated perspective may accelerate the translation of FM biology into improved risk assessment tools and novel therapeutic strategies.

## 1. Introduction

Fibrin monomer (FM) is a transient product of blood coagulation produced upon thrombin cleavage of fibrinogen. Under normal conditions, FM rapidly self-associates to form protofibrils, fibrin strands, and ultimately the fibrin meshwork that stabilizes thrombi [1,2]. However, FM that escapes immediate polymerization can bind to fibrinogen or fibrin degradation products (FDP) and form soluble fibrin monomer complexes (SFMC) [3]. As an upstream product in the coagulation cascade, FM appears during the initial stages of thrombin-mediated fibrin formation. As a result, FM rises early in prethrombotic states, often preceding detectable increases in D-dimer and other fibrin degradation products [4]. Clinically, this temporal priority enables potential use of SFMC as a prethrombotic marker or an early indicator of thrombotic risk. Growing evidence indicates that FM could be useful in a variety of clinical conditions, including disseminated intravascular coagulation, venous thromboembolism, myocardial infarction, stroke and perioperative thrombosis [5,6,7]. FM is a promising thrombotic biomarker not only for diagnosing prethrombotic states and ongoing thromboses but also for risk assessment of incipient thrombotic complications [6].

Beyond its diagnostic utility, FM has proven to be an active regulator of haemostasis. Following thrombin cleavage of fibrinogen, FM represents a critical intermediate that retains some fibrinogen-like properties while acquiring novel binding characteristics [8]. The transient presence of fibrin monomers at sites of active thrombin generation suggests unique regulatory roles during early stages of thrombus formation. Fibrin modulates thrombotic processes through interactions with platelets and coagulation factors [9]; it influences both procoagulant and anticoagulant pathways [10] and engages in inflammation and tissue repair [11]. These interactions involve both monomeric and polymerized forms of fibrin. Understanding the specific contributions of FM, as distinct from fibrinogen and polymerized fibrin, remains an important frontier in haemostasis research.

**Figure 1:**
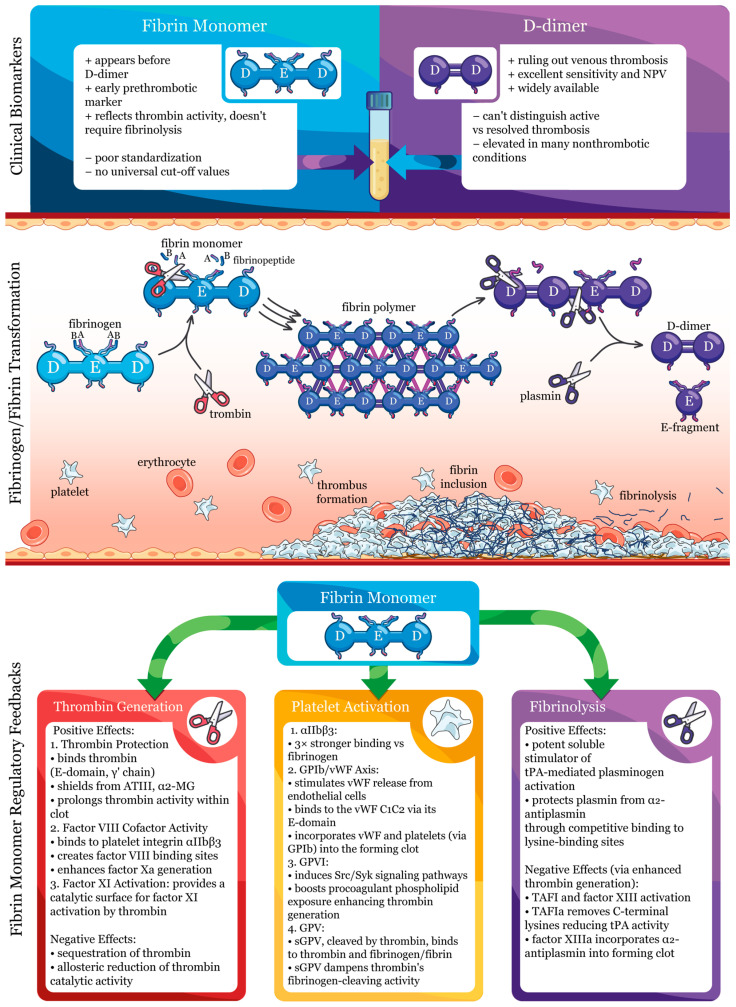
Simplified scheme of fibrin monomer’s involvement in the regulation of haemostasis. Abbreviations: α2-MG, α2-macroglobulin; ATIII, antithrombin III; NPV, negative predictive value; TAFI, thrombin-activatable fibrinolysis inhibitor; tPA, tissue-type plasminogen activator; vWF, von Willebrand factor.

The dual identity of FM, as both a mechanistic participant in haemostatic regulation and a sensitive diagnostic indicator of prothrombotic states, represents the central theme of the present review and defines its intended readership: researchers investigating molecular mechanisms of haemostasis and clinicians seeking evidence-based guidance on FM biomarker applications. The present review summarizes the current knowledge on FM’s mechanistic roles in haemostasis regulation and the accumulated evidence of its utility as a clinical biomarker across various thrombotic conditions. By synthesizing recent advances from both basic and clinical research, this review aims to provide a broader perspective to stimulate the development of new ideas and clinical hypotheses for future investigations. The multifaceted roles of fibrin monomer in haemostasis are schematically summarized in Figure 1, reflecting FM’s regulatory feedback on thrombin generation, platelet activation, and fibrinolysis, as well as its utility as a clinical biomarker compared to D-dimer.

## 2. Involvement of Fibrin Monomer in the Regulation of Haemostasis

### 2.1. Multiple Roles of Fibrinogen and Fibrin

Fibrin is the most essential component of the blood clot, making up its structural scaffold [1,2]. Fibrin is formed from fibrinogen, which is abundantly present in human blood, by the key coagulation enzyme—thrombin [12]. Generation of thrombin is tightly regulated by an intricate network of biochemical and cellular interactions [13]. Upon sufficient stimulation, self-accelerated thrombin generation occurs, leading to a rapid conversion of fibrinogen into fibrin monomer and its subsequent polymerization [14].

Thrombin cleaves fibrinogen, releasing fibrinopeptides A and B (FpA and FpB) [15]. Initially, the release of FpA enables so-called “knob-hole” interactions between individual fibrin monomers. These interactions lead to spontaneous half-staggered polymerization, forming fibrin oligomers and protofibrils. Subsequent release of FpB changes the conformation of αC regions of fibrin and facilitates lateral aggregation of protofibrils into thicker fibers. Eventually, the polymerization process leads to the formation of a branched fibrin network [16]. In addition to converting fibrinogen to fibrin, thrombin also regulates a clot’s stability by activating coagulation factor XIII (FXIII) [17]. In its active form, FXIIIa covalently crosslinks α and γ chains of fibrin, increasing the stability of the forming clot [18]. Notably, fibrin itself exerts an inhibitory effect on thrombin generation, providing negative regulatory feedback (so-called anti-thrombin I activity) [19].

Fibrinogen and fibrin are directly involved in cellular regulation of haemostasis as well [9]. They take part in the processes of platelet activation, aggregation and adhesion by interacting with different platelet receptors and shear-sensitive protein von Willebrand Factor (vWF) [20]. Thrombus consolidation is governed by clot retraction, which results from platelets imposing contractile forces on the fibrin mesh [21]. Recently it has been established that this process largely depends on FXIII-mediated crosslinking, which regulates the retention of red blood cells within the clot [22].

Finally, the fibrin network is a substrate for clot lysis performed by plasmin. The fibrinolytic system, aimed at recanalization of the vessel lumen and restoring blood circulation, is regulated by a variety of plasmatic and cellular factors [23,24]. Notably, fibrin itself acts not only as a substrate for plasmin but also as one of the main catalyzers for plasmin generation. The efficacy of tissue-type plasminogen activator (tPA) increases by up to three orders of magnitude in the presence of fibrin [25].

This brief overview shows that fibrin is not only the main structural block of forming blood clots but is also involved on multiple levels in plasmatic and cellular regulation of haemostasis. Structure of the fibrin network determines the physical properties of the forming clot—its stability, elasticity and fibrinolytic resistance [26]. Eventually all these properties have a great impact on the clinical outcomes of thrombotic complications [27], as well as on the ability to stop bleeding after vascular injury [28].

### 2.2. Blood Coagulation

The historical designation of fibrin as “antithrombin I” emerged from early observations that fibrin removes thrombin from solution through adsorption, suggesting a purely anticoagulant function [29]. However, contemporary evidence reveals that fibrin possesses a sophisticated dual regulatory role, functioning simultaneously as both a thrombin sequestrant and an active procoagulant amplifier of haemostasis [10]. This apparent paradox reflects the complex molecular mechanisms through which fibrin monomers and polymers modulate thrombin activity and coagulation factor dynamics.

The binding of thrombin to fibrin occurs at specific sites [30,31]. The E domain contains low-affinity binding sites engaging thrombin exosite I, while the alternatively spliced γ’ chain variant (present in ~10–15% of fibrinogen molecules) provides high-affinity binding through thrombin exosite II. Fibrin formation results in both physical sequestration of thrombin and allosteric reduction in its catalytic activity, particularly through exosite II engagement [19,32]. Thrombin bound to fibrin is partly protected from inhibitors like antithrombin III and α2-macroglobulin, especially within the confines of a clot [33,34]. Limited diffusion of large inhibitor molecules into the fibrin mesh means that thrombin can remain active longer when bound to fibrin fibers [34]. This protection mechanism enables sustained thrombin activity within a forming clot despite circulating inhibitors. In this way, fibrin monomer/polymer plays a dual role: by absorbing thrombin it reduces immediate downstream proteolysis of fibrinogen in the fluid (negative feedback), but by holding thrombin near fibrin and shielding it from inhibitors, it prolongs thrombin’s procoagulant activity within the clot (positive feedback).

Factor XI interactions with fibrin create additional amplification mechanisms [35]. Fibrin surfaces provide optimal scaffolds for thrombin-mediated Factor XI activation, establishing positive feedback that generates additional thrombin through the intrinsic pathway. This mechanism is able to generate substantial additional thrombin within fibrin clots, protecting them from fibrinolysis through thrombin-activatable fibrinolysis inhibitor (TAFI) [35,36]. Epidemiological evidence has confirmed that elevated FXI levels correlate with venous thrombosis risk [37]. These findings have spurred development of Factor XI inhibitors that may uncouple antithrombotic effects from bleeding risk [38].

Recent work has identified fibrin as a critical cofactor for factor VIII (FVIII) function on activated platelets [39]. When soluble fibrin binds to the platelet αIIbβ3 integrin, it creates additional FVIII binding sites and enhances factor Xa activity [39]. Remarkably, FVIII binding to thrombin-stimulated platelets occurs predominantly through platelet-bound fibrin rather than phosphatidylserine exposure. Phosphatidylserine accounts for less than 10% of binding sites, despite traditional models emphasizing phosphatidylserine as the primary membrane determinant. Factor VIII mutants defective in phosphatidylserine binding retained 45% of their platelet binding capacity, confirming the existence of non-phosphatidylserine binding sites [39]. These findings suggest that a fibrin–FVIII interaction may create a positive feedback mechanism, wherein initial fibrin formation amplifies FVIII activity, generating additional thrombin and thus more fibrin, thereby localizing and intensifying coagulation at sites of vascular injury.

Fibrin monomer represents an active coagulation regulator, not merely a structural precursor awaiting polymerization. Monomeric fibrin protects thrombin from heparin-antithrombin III inactivation [40] and provides functional thrombin binding sites [10], maintaining thrombin activity at sites of fibrin formation. Most significantly, soluble fibrin functions as a critical cofactor for factor VIII on activated platelets, creating FVIII binding sites and enhancing factor Xa generation [39]—establishing positive feedback wherein initial fibrin formation amplifies thrombin production and subsequent fibrin generation. Additionally, fibrin provides scaffolds for thrombin-mediated factor XI activation [35] and engages platelet receptors to trigger procoagulant phospholipid exposure that enhances tenase and prothrombinase activities [9]. Polymerization intensifies these functions through increased binding site density and spatial organization. This hierarchical regulatory architecture—from monomer-initiated amplification to polymer-enhanced stabilization—ensures robust, localized haemostasis under physiological conditions but drives pathological thrombosis when dysregulated.

### 2.3. Fibrinolysis

Fibrin plays a central regulatory role in the fibrinolytic system by coordinating plasminogen activation while protecting plasmin from rapid inhibition. Through multiple mechanisms involving lysine–binding-site interactions and conformational changes, fibrin creates a localized proteolytic microenvironment essential for controlled clot dissolution. This regulatory system achieves a remarkable catalytic enhancement (up to three orders of magnitude) while maintaining spatial specificity to prevent systemic fibrinolysis [41]. The conversion of fibrinogen to fibrin monomer through thrombin-mediated cleavage of fibrinopeptides A and B triggers conformational changes that expose cryptic binding sites for tissue plasminogen activator (tPA) and plasminogen, which is fundamental to fibrin’s regulatory function. Polymerized fibrin enhances the catalytic efficiency of plasminogen activation by tPA approximately 300- to 600-fold compared to reactions in solution, representing one of the most potent cofactor effects in haemostasis [42].

The formation of a ternary fibrin-tPA-plasminogen complex colocalizes enzyme and substrate on the fibrin surface. This spatial organization dramatically enhances plasmin generation in the presence of fibrin [43]. The resulting fibrin specificity ensures that plasmin generation occurs predominantly at sites of fibrin deposition rather than systemically. The αC-domain exhibits particularly high affinity for both tPA and plasminogen and undergoes conformational changes from a compact state in fibrinogen during fibrin formation and polymerization [25]. When polymeric fibrin is formed, D-D/E interactions expose and activate multiple cryptic binding sites in various regions, including the αC-domains. This conformational plasticity, combined with the ordered fibrin surface, concentrates and orients plasminogen and tPA in close proximity, creating an enhanced catalytic environment that is more effective for plasminogen activation than isolated fibrin domains.

This enhancement is further increased as plasmin initiates fibrin degradation, generating additional C-terminal lysine binding sites. Initial fibrin possesses limited exposed C-terminal lysines, but plasmin degradation generates new high-affinity binding sites, accelerating fibrinolysis by enhancing recruitment of both plasminogen and tPA to the clot surface [44]. This positive feedback mechanism is counter-regulated by TAFI, which removes C-terminal lysines from partially degraded fibrin, thereby reducing plasminogen binding and slowing fibrinolysis. The TAFI system provides a crucial link between the coagulation and fibrinolytic pathways, allowing thrombin generation to modulate subsequent clot dissolution [45,46]. Additionally, thrombin-dependent clot protection is facilitated by Factor XIIIa, which covalently crosslinks fibrin α-chains through αC-domain interactions and incorporates α2-antiplasmin into the fibrin network, thereby enhancing both structural stability and fibrinolytic resistance [18,47].

Notably, fibrin monomer and fibrin provide significant protection of plasmin from α2-antiplasmin. This protection mechanism operates through competitive binding. C-terminal lysines present in both fibrin and α2-antiplasmin compete for the same lysine-binding sites on plasmin’s kringle domain. When fibrin occupies these sites, it blocks the initial non-covalent binding of α2-antiplasmin to plasmin—thereby preventing the subsequent irreversible covalent complex formation [48].

Dysregulation of these regulatory mechanisms contributes to pathological conditions, such as the formation of dense fibrin networks with thin fibers and small pores that exhibit hypofibrinolysis. These structural and functional abnormalities associate with venous thromboembolism, myocardial infarction, and stroke and represent important risk factors for thrombotic events and therapeutic resistance [49].

Polymerized fibrin regulates its own lysis by confining enzymatic processes to the forming clot through higher binding site density and optimal spatial organization of cofactor surfaces. Fibrin monomer, in turn, also demonstrates significant regulatory capacity independently of polymerization. It has been established that fibrin monomer is the most potent soluble stimulator of tPA-mediated plasminogen activation among all fibrin(ogen) derivatives [50]. It has also been demonstrated that fibrin monomer substantially protects plasmin from α2-antiplasmin inhibition through competitive binding at lysine-binding sites, reducing the inhibition rate approximately 20-fold [51]. Furthermore, soluble fibrin degradation products, particularly (DD)E complex generated during clot lysis, can potentiate tPA-induced fibrinogenolysis [52]. This finding might have clinical implications: elevated SFMC levels during thrombolytic therapy may promote systemic lysis and hemorrhagic complications, as these soluble complexes can activate plasminogen at sites distant from the primary thrombus, potentially compromising the hemostatic function.

While the aforementioned findings emphasize FM’s potential role in bleeding complications, emerging experimental evidence suggests its therapeutic applications for hemorrhage control. Studies investigating exogenous FM administration in experimental trauma have demonstrated significant hemostatic efficacy with favorable safety profiles [53], positioning FM as a candidate for development as a systemic hemostatic agent. Unlike antifibrinolytic therapies that globally suppress fibrinolysis, FM-based approaches may leverage endogenous regulatory pathways to achieve localized haemostasis. However, translation from experimental models to clinical applications requires rigorous evaluation of dosing strategies, pharmacokinetics, and long-term safety in human subjects.

### 2.4. Platelet Interactions

**Figure 2:**
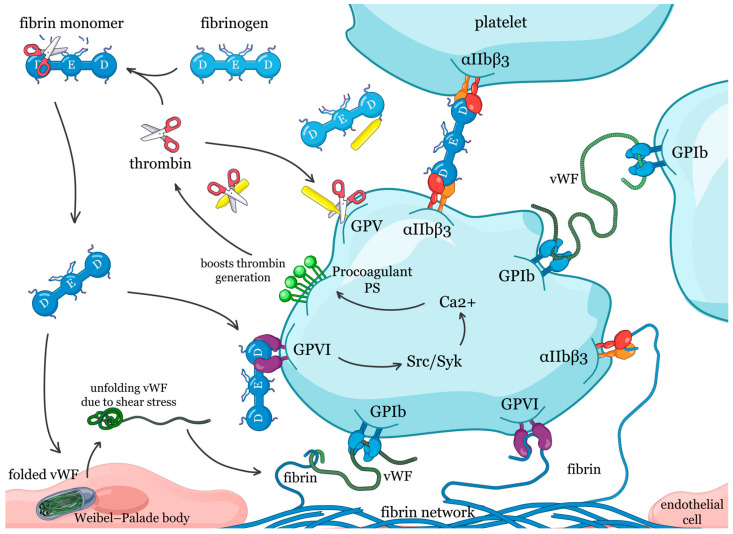
Schematic representation of platelet–fibrin-monomer interactions.

Fibrin monomer engages multiple platelet receptors, including integrin αIIbβ3, GPVI, the GPIb-vWF axis, and GPV (Figure 2). The following sub-sections detail the discovery and molecular mechanisms of these receptor-mediated interactions.

#### 2.4.1. Early Studies of the Platelet–Fibrin-Monomer Interactions

Early works investigating how fibrin intermediates influence platelet function appeared in the 1970s. Pioneering studies established that soluble fibrin monomer complexes directly induce platelet aggregation [54]. Subsequent work showed that polymerizing fibrin was particularly effective at inducing aggregation [55], with platelets interacting preferentially with intermediate polymerization products in a calcium-dependent manner [56].

These findings were refined when fluorescence-labeled fibrin studies demonstrated that platelets interact with fibrin only after activation, requiring pseudopod formation for binding [57]. This clarified that active molecular binding differs from passive physical trapping in fibrin networks. The molecular basis was elucidated using site-directed mutagenesis, revealing that the fibrinogen γ-chain carboxyl-terminal region (γ400–411) is essential for platelet aggregation via the αIIbβ3 integrin receptor [58,59].

Flow chamber studies under physiological shear provided crucial mechanistic insights. Conversion of immobilized fibrinogen to fibrin monomer dramatically enhanced platelet adhesion and aggregate formation, with fibrin protofibrils exhibiting superior binding compared to both fibrinogen and fibrin monomer [60]. This progression reflects how fibrinopeptide cleavage exposes new binding epitopes and creates multivalent binding opportunities through oligomerization.

Collectively, these studies established fibrin monomer as a transitional species with enhanced platelet-binding properties compared to fibrinogen. Fibrinopeptide cleavage and conformational changes optimizing γ-chain presentation create a highly thrombogenic substrate, positioning fibrin monomer as both a biomarker of active coagulation and a mechanistic participant in thrombus stabilization.

#### 2.4.2. Integrin αIIbβ3

Integrin αIIbβ3 is the most abundant receptor on platelets and serves as the primary mediator of platelet aggregation and clot retraction. The interaction between αIIbβ3 and fibrin(ogen) involves multiple recognition sites that contribute differently to various hemostatic processes [59]. The γ-chain C-terminal sequence γ400–411 contains the primary binding site for soluble fibrinogen and is constitutively exposed in both fibrinogen and fibrin monomer [58]. Two RGD motifs located in the Aα chain (Aα95–97 and Aα572–574) remain cryptic in soluble fibrinogen but become accessible upon its surface immobilization or conversion to fibrin [58,61]. Additionally, the P3 region (γ370–383) is fibrin-specific and exposed only upon fibrinogen-to-fibrin transformation, mediating clot retraction and platelet adhesion to fibrin [62].

Single-molecule force spectroscopy studies have revealed a hierarchy in binding strength: polymeric fibrin > fibrin monomer > fibrinogen [63,64]. Using optical trap-based measurements, fibrin monomer demonstrated a 3-fold higher cumulative binding probability to αIIbβ3 compared with fibrinogen, despite identical surface densities [63]. The average cumulative binding probability for fibrin monomer reached approximately 30% throughout the force range, compared to 11.5% for fibrinogen [63]. Moreover, fibrin monomer exhibited reduced sensitivity to competitive inhibition by the γC-dodecapeptide and cyclic RGD peptides compared with fibrinogen, suggesting that additional cryptic binding sites become exposed during fibrin monomer formation [63]. Polymeric fibrin exhibited even stronger interactions, with mechanical stability following the established hierarchy [64].

The physiological relevance of FM-platelet interactions is supported by soluble fibrin in plasma, which consists predominantly of des-AA fibrin monomers along with a minor component of protofibril/fibrinogen clusters [65,66]. When adsorbed onto hydrophobic surfaces, these des-AA monomers spontaneously initiated protofibril assembly and generated three-dimensional fiber networks [65]. These fibrin-monomer-derived fibers exhibited robust platelet binding through exposed αIIbβ3 recognition motifs, particularly within the Aα518–584 and γ86–411 regions [65]. Soluble fibrin-enriched preparations accelerated thrombin-induced polymerization, increased clot stiffness, and generated fibers tightly anchored to underlying surfaces [65,66]. The spontaneous fiber-forming capacity of FM, combined with its ability to link platelets and erythrocytes, indicates that soluble fibrin represents a hypercoagulable component amplifying hemostatic responses [66].

Collectively, these findings establish that fibrin monomers represent a distinct αIIbβ3 ligand with enhanced binding properties compared to fibrinogen, facilitating the critical transition from initial platelet aggregation to stable thrombus formation during haemostasis. During haemostasis, thrombin generation at sites of vascular injury simultaneously activates platelets and converts fibrinogen to fibrin monomer [67]. This creates a dynamic environment where αIIbβ3 interactions progressively shift from fibrinogen to fibrin monomer and ultimately to polymerized fibrin. Fibrin monomer serves as a critical intermediate in this transition, providing stronger and more stable platelet–ligand bonds than fibrinogen while maintaining the solubility necessary for initial incorporation into developing thrombi. The binding strength hierarchy has profound implications for thrombus stability and organization. Stronger αIIbβ3–fibrin interactions, particularly with polymerized fibrin in the thrombus core, contribute to clot consolidation and resistance to disruption [21,68]. The hierarchical organization of thrombi, with a tightly packed core of fully activated platelets surrounded by a less stable shell, depends partly on differential ligand engagement and local agonist gradients [67,68].

#### 2.4.3. Glycoprotein Ib & von Willebrand Factor

The interplay between fibrin monomer, von Willebrand factor (vWF), and the platelet receptor glycoprotein Ib (GPIb) represents a crucial regulatory mechanism in haemostasis and thrombosis. Early investigations demonstrated that fibrin induces rapid vWF release from endothelial cells [69]. It was shown that fibrin monomer is a crucial stimulator of this process and that cleavage of FpB and exposure of β15–42 sequence in fibrin β-chain are required for vWF secretion [70]. Concurrently, it was discovered that fibrin monomer enhances platelet vWF binding to GPIb through direct fibrin–glycocalicin interactions on thrombin-stimulated platelets [71].

Subsequent studies established that platelet adhesion to fibrin in flowing blood involves complex tripartite interactions. Under flow conditions, GPIb, vWF, and αIIbβ3 all contribute to platelet adhesion to fibrin, with their relative importance varying depending on shear rate [72]. At high shear rates, the GPIb-vWF interaction becomes particularly critical, accounting for up to 70% of platelet adhesion [4]. Importantly, vWF does not bind directly to immobilized fibrinogen or fully polymerized fibrin surfaces but requires platelet-mediated bridging via GPIb for its adhesion to these surfaces [73].

Beyond their role in adhesion, these GPIb–vWF–fibrin interactions also regulate platelet procoagulant activity and thrombin generation. Studies by Béguin et al. demonstrated that fibrin-mediated enhancement of thrombin generation requires GPIb [74]. Specifically, GPIb blockade eliminated the fibrin enhancement while leaving basal thrombin generation intact [74]. These studies identified two distinct vWF-dependent mechanisms contributing to platelet procoagulant activity: a fibrin-independent pathway mediated by αIIbβ3, and a fibrin-dependent pathway mediated by GPIb. Importantly, patients with Bernard–Soulier syndrome, who lack functional GPIb, exhibit defective fibrin-dependent thrombin generation, providing a mechanistic explanation for their bleeding tendency [75].

The thrombin-binding site on GPIb is essential for platelet procoagulant activity and phosphatidylserine exposure [76], creating a positive feedback loop wherein GPIb-bound thrombin generates additional fibrin monomers that amplify fibrin-dependent responses. This finding prompted debate regarding the mechanism by which polymerizing fibrin influences platelet behavior. One study proposed that fibrin actively mediates GPIb-dependent platelet aggregation through receptor signaling [77], whereas another demonstrated that thrombin-induced fibrin conversion results in rapid platelet entrapment independent of GPIb activation [78]. Subsequent work demonstrated that, even under low-shear conditions, the GPIb-V-IX complex and vWF actively participate in platelet activation-dependent fibrin formation, anchoring fibrin networks to activated platelet surfaces [79], though the precise interplay between these processes remains an area of active investigation.

Studies using deletion mutants and fragment competition identified the high-affinity fibrin-binding site in the C1C2 domain of vWF, distinct from the GPIb-binding A1 domain [80]. Subsequently, surface plasmon resonance and ellipsometry studies revealed that vWF does not bind immobilized fibrinogen or fully polymerized fibrin but binds specifically to fibrin monomers during active fibrinogen-to-fibrin conversion in the presence of thrombin [81]. This binding involves the vWF C1C2 domain interacting with the fibrin E-domain at fibrinopeptide cleavage sites and proceeds via FXIIIa-independent non-covalent binding followed by covalent crosslinking [81]. These findings demonstrate that vWF incorporation into fibrin networks occurs specifically during the fibrinogen-to-fibrin conversion through direct monomer binding [81], while vWF association with fully polymerized fibrin surfaces requires platelet-mediated bridging via GPIb and αIIbβ3 [73,81].

Collectively, these findings establish fibrin monomer as a critical regulator orchestrating vWF-platelet interactions and integrating primary haemostasis with fibrin network formation. Understanding the role of fibrin monomer in the vWF-GPIb axis provides mechanistic insight into bleeding complications observed in Bernard-Soulier syndrome and von Willebrand disease. Recent studies using engineered microvessels lined with human endothelial cells have confirmed that endothelial-derived vWF colocalizes with polymerizing fibrin and accelerates clot formation under flow conditions, providing an experimental platform for further investigation of these interactions in a more physiological setting [82].

#### 2.4.4. Glycoprotein VI

For many years, glycoprotein VI (GPVI) was recognized exclusively as the primary platelet receptor for collagen. This understanding changed fundamentally in 2015 when two landmark studies simultaneously demonstrated that GPVI also recognizes fibrin [83,84]. It was shown that immobilized fibrin (but not fibrinogen) activates GPVI through Src and Syk phosphorylation cascades, with both monomeric and polymerized fibrin binding to the GPVI ectodomain [83]. Critically, GPVI binding to polymerized fibrin promotes drastic amplification of thrombin generation and enhances platelet recruitment to fibrin-rich clots by up to 85%, establishing a positive feedback loop linking platelet activation to coagulation amplification [84].

Whether GPVI must be dimeric to bind fibrin sparked intense debate, as comprehensively reviewed by Slater et al. [85]. Early studies reported that fibrin binds to monomeric GPVI (Kd = 302 nM), contrasting with collagen’s established preference for dimeric GPVI [86]. However, when binding to fibrin clots, dimeric GPVI exhibits specific, high-affinity binding, while monomeric GPVI demonstrates only negligible or nonspecific binding [87], suggesting that fibrin formation alters the binding specificity. Surface plasmon resonance measurements revealed that both monomeric and dimeric GPVI bind fibrinogen, with dimeric GPVI exhibiting approximately 50-fold higher affinity through avidity effects characterized by dramatically slower dissociation kinetics [88]. Species-specific differences further complicated interpretations, as human but not mouse GPVI binds to immobilized fibrinogen and supports full platelet spreading through Syk-dependent signaling [89].

Molecular characterization revealed that both the αC-region and the D-region of fibrin serve as binding sites for GPVI [88]. Initial studies localized GPVI binding specifically to the D-region [86,90]. Importantly, GPVI-binding epitopes in the D-region appear cryptic in intact fibrinogen but become exposed following fibrinopeptide cleavage during fibrin formation [87]. Subsequently, it was shown that the αC-region, specifically its C-terminal globular domain, exhibits much higher binding affinity for GPVI [88]. These findings support a model wherein the αC-region provides initial high-affinity recognition, while fibrin polymerization brings multiple binding sites (both αC and D-regions) into proximity to cluster GPVI and triggers platelet activation [88,90].

GPVI–fibrin interactions extend beyond adhesion alone. Research demonstrated nonredundant roles for GPVI and integrin αIIbβ3 in fibrin-mediated microthrombus formation, with GPVI specifically mediating Ca^2+^ signaling and platelet secretion [91]. GPVI–fibrin interaction significantly increases procoagulant platelet activity, creating denser, less porous clots with a prothrombotic phenotype [92]. The thrombin generation amplification represents a critical physiological mechanism: GPVI binding to fibrin triggers platelet activation, procoagulant phospholipid exposure, enhanced thrombin generation, and further fibrin formation, establishing a self-reinforcing positive feedback cycle [84].

However, the physiological relevance of these observed effects remains a subject of debate. Several studies have challenged whether GPVI functionally binds fibrin under physiological conditions, demonstrating that plasma proteins—particularly albumin intercalating within fibrin fibers—shield GPVI-activating epitopes, distinguishing “pure fibrin” from physiologically relevant plasma fibrin [93,94]. These discrepancies likely reflect differences in both experimental methodology and reagent quality: fibrin preparation methods, GPVI construct variations, surface immobilization artifacts, and fibrinogen integrity—particularly proteolytic degradation of the critical αC-region [85,95].

Regarding fibrin monomer, evidence indicates that polymerization is not strictly required for GPVI binding, as non-polymerizing fibrin mutants bind GPVI with similar affinity to native fibrin [88]. On the other hand, this finding was made using immobilized fibrin [88], while in solution, platelet activation required fibrin polymerization and was blocked by GPRP [83]. Paradoxically, soluble fibrin oligomers cause acquired GPVI signaling dysfunction by binding and desensitizing the receptor, potentially explaining platelet hypofunction in trauma-induced coagulopathy [96]. Understanding these complex interactions holds therapeutic promise, as GPVI blockade could potentially prevent pathological thrombosis while preserving primary haemostasis with minimal bleeding risk compared to conventional antiplatelet agents [97]. This positions GPVI as an attractive target for next-generation antithrombotic strategies that specifically interrupt the platelet-fibrin positive feedback loop while maintaining initial haemostatic plug formation.

#### 2.4.5. Glycoprotein V

Platelet Glycoprotein V (GPV) is recognized primarily as a component of the GPIb-IX-V receptor complex and a substrate for thrombin proteolysis during platelet activation [98]. Studies using GPV-deficient mice demonstrated that platelets lacking GPV exhibited hyperresponsiveness to thrombin at low concentrations, suggesting GPV acts as a negative regulator of thrombin-induced platelet activation [99]. However, the physiological function of soluble GPV (sGPV) released after proteolytic cleavage of GPV by thrombin remained unclear [98].

A recent study [100] revealed that sGPV modulates the formation of fibrin through a dual interaction with both thrombin and fibrin. Using a genetic mouse model unable to shed GPV alongside GPV-deficient mice, the authors demonstrated accelerated fibrin formation and enhanced thrombosis when GPV shedding was prevented. Biochemical experiments confirmed that sGPV binds thrombin and dampens its fibrinogen-cleaving activity. In the presence of sGPV, fibrin networks displayed altered architecture with thicker, less frequent fibers and reduced structural definition. Super-resolution microscopy revealed that sGPV accumulates in fibrin-rich, platelet-free regions of thrombi. Importantly, sGPV localized to fibrin regardless of the clot-inducing enzyme (thrombin or batroxobin), demonstrating direct interaction of sGPV with fibrin independent of thrombin–GPV complex formation [100]. Altogether, these results revealed a previously unknown negative feedback that prevents excessive thrombin-mediated fibrin generation at sites of platelet deposition. Specific binding sites for sGPV on monomeric and polymeric fibrin are as yet unknown, but their identification may contribute to a better understanding of this novel regulatory mechanism.

### 2.5. Platelet–Tumor Cell Interactions

Fibrin monomer may also enhance platelet interactions with other cell types, such as circulating tumor cells. A notable example is platelet–cancer embolus formation in metastasis [101,102]. Melanoma cells pretreated with fibrin monomer bind significantly more platelets than those pretreated with fibrinogen [101]. This interaction, mediated via platelet integrin αIIbβ3 and tumor cell CD54 (ICAM-1), enhanced experimental lung metastasis by 65% [102]. Subsequently, the same group demonstrated that soluble fibrin inhibits monocyte and lymphocyte adherence and cytotoxicity against tumor cells [103,104]. While falling outside traditional haemostasis, these findings underscore fibrin monomer’s pathophysiologic role in bridging platelets to tumor cells and modulating immune response.

### 2.6. Leukocytes

Fibrinogen and fibrin serve as critical molecular links between coagulation activation and inflammatory responses [105]. The leukocyte integrin αMβ2 (Mac-1, CD11b/CD18) has emerged as a key receptor mediating these interactions, with fibrin binding sites localized to specific motifs within the fibrinogen γ-chain [106]. Critically, soluble fibrinogen is a relatively poor ligand for αMβ2, whereas immobilized or polymerized fibrin exhibits high-affinity binding. This difference in binding capacity results from a cryptic binding motif that remains inaccessible in soluble fibrinogen but becomes exposed upon surface immobilization or conversion into fibrin polymer [107].

The physiological importance of fibrin engagement by αMβ2 has been definitively demonstrated through genetic studies. Mice bearing a targeted γ-chain mutation (Fibγ390–396A) that eliminates αMβ2 binding while preserving hemostatic function showed dramatically impaired neutrophil activation and antimicrobial capacity [108]. Despite maintaining normal clotting parameters, these mice exhibited severely compromised leukocyte effector functions, including defective bacterial clearance and blunted inflammatory responses, confirming that fibrin-αMβ2 engagement is essential for innate immunity [108].

Interestingly, fibrin networks formed under physiological flow conditions demonstrate enhanced adhesive properties for neutrophils compared to static fibrin structures [109]. Flow-formed fibrin supports up to threefold greater neutrophil adhesion through CD11b/CD18-dependent mechanisms. Furthermore, the same study showed that fibrinogen can function as an inhibitor of neutrophil adhesion to the fibrin surface [109].

Beyond αMβ2-mediated interactions, fibrin monomer engages the very low-density lipoprotein receptor (VLDLR), which modulates fibrin-dependent transendothelial migration of leukocytes [110]. The VLDLR-binding sites within fibrin βN-domains are cryptic in fibrinogen but become accessible upon fibrin formation. Notably, unlike αMβ2, VLDLR binds soluble fibrin monomers. This provides a unique mechanism by which FM can modulate leukocyte trafficking during inflammation even before fibrin polymerization occurs [110].

## 3. Fibrin Monomer as a Clinical Biomarker

Clinical measurements of FM reflect circulating soluble fibrin species (predominantly SFMC) rather than free monomeric fibrin. Various assay methodologies detect different molecular complexes but are collectively reported as FM levels or concentrations. To maintain consistency with the clinical literature and avoid confusion, “FM” throughout this section refers to these measured parameters, while Section 2 addressed FM as the monomeric molecular entity.

### 3.1. Myocardial Infarction

Acute myocardial infarction (AMI) is caused by thrombotic occlusion of coronary arteries, making fibrin monomer measurement particularly relevant for detecting active thrombotic processes underlying cardiac ischemic events. Investigations of FM in AMI have demonstrated consistent elevation during acute phases. Early studies established that FM levels are markedly elevated in patients with unstable coronary artery disease and AMI compared to those with stable angina or healthy controls [111,112]. Elevated FM concentrations reflect active thrombin generation at sites of coronary plaque disruption, providing a molecular signature of ongoing thrombotic processes. In AMI patients, soluble fibrin levels demonstrated significant associations with clinical severity, with higher concentrations observed in those experiencing complications [112,113].

The prognostic significance of FM measurement in AMI has been demonstrated through multiple prospective studies. In 293 patients with acute anterior myocardial infarction, elevated FM levels on days 2 and 7 post-event independently predicted 3-month mortality, with non-survivors showing median FM levels of 1.8 μg/mL versus 0.4 μg/mL in survivors [113]. Mortality risk demonstrated a significant association with FM levels expressed in quartiles, ranging from 1.4% in the lowest to 15.1% in the highest quartile. FM levels correlated significantly with enzymatic infarct size and were elevated in patients developing congestive heart failure [113].

Additional studies have validated FM’s diagnostic and prognostic utility across diverse AMI populations. In young men with AMI (<45 years), soluble fibrin emerged as the strongest independent predictor of myocardial infarction on multivariate analysis, surpassing traditional risk factors [114]. Investigation of treatment modalities revealed that primary percutaneous coronary intervention produced minimal FM elevation compared to substantial increases with thrombolysis, distinguishing mechanical and pharmacological reperfusion [115]. Long-term follow-up studies confirmed that FM levels predicted recurrent ischemic events, with elevated concentrations identifying patients requiring intensified secondary prevention strategies [116].

FM demonstrated particular utility in specific clinical contexts. Analysis of coronary thrombotic events revealed that FM levels effectively identified patients at risk for adverse outcomes [117]. Studies examining FM in conjunction with traditional biomarkers showed that FM enhanced early and accurate AMI diagnosis [118]. More recent multicenter analyses incorporating broader cardiovascular disease cohorts, including those with atrial fibrillation and heart failure, confirmed FM’s predictive value for cardiovascular and cerebrovascular events, extending its applicability beyond isolated coronary syndromes [119,120]. Contemporary studies demonstrated that soluble fibrin measurement, particularly when combined with D-dimer assessment, provides superior diagnostic accuracy for thrombotic diseases, including AMI, with notably low FDP/SF ratios distinguishing arterial from venous thrombosis [121].

Collectively, these investigations establish FM as a sensitive biomarker for detecting hypercoagulable states in AMI, enabling enhanced risk stratification when integrated with conventional clinical parameters. FM demonstrates particular value as an independent mortality predictor in the acute phase and maintains prognostic significance during long-term follow-up. Integrating FM measurement into clinical algorithms could enhance risk stratification, particularly for identifying patients who may benefit from intensified antithrombotic therapy. However, widespread clinical implementation requires standardization of assay methodologies and establishment of universally applicable cut-off values.

### 3.2. Atrial Fibrillation

Atrial fibrillation (AF) represents the most common sustained cardiac arrhythmia and is characterized by a prothrombotic state that significantly increases the risk of thromboembolic complications and cardiovascular mortality. The hypercoagulable state in AF results from alterations in haemostasis, endothelial dysfunction, and abnormal blood flow patterns, particularly within the left atrial appendage (LAA) [122].

Several studies have investigated the relationship between FM levels and thrombotic risk in AF patients. In acute ischemic stroke, FM levels were markedly elevated in patients with LAA thrombus compared to those without (88 ± 52 vs. 14 ± 9 μg/mL), with FM emerging as an independent predictor of LAA thrombus formation [123]. Additionally, FM levels correlated with abnormal LAA flow patterns, suggesting that elevated FM reflects the hypercoagulable state associated with atrial mechanical dysfunction [123]. Among acute stroke patients with AF, FM levels were significantly higher than in non-AF patients and showed positive correlations with plasmin-α2-antiplasmin complex, further supporting the role of FM in AF-associated thrombogenesis [124].

Regarding its prognostic value, the Murcia Atrial Fibrillation Project demonstrated that FM > 12 μg/mL was significantly associated with increased risk of adverse cardiovascular events, cardiovascular mortality, and all-cause mortality in AF patients receiving vitamin K antagonists [119]. However, FM was not associated with ischemic stroke risk. When added to the CHA2DS2-VASc score, FM improved predictive performance for cardiovascular events and mortality, though clinical usefulness remained limited [119,125]. Conversely, another study found that FM levels during oral anticoagulation did not predict subsequent thromboembolic events in permanent AF [126].

In the ANAFIE (All Nippon Atrial Fibrillation In the Elderly) registry of elderly Japanese patients with AF, positive FM was associated with increased major bleeding and cardiovascular events in the warfarin group, while D-dimer and thrombin-antithrombin complex showed stronger associations in the direct oral anticoagulant group [127]. Intracardiac blood sampling studies revealed no significant AF-specific alterations in FM levels between AF patients and controls during catheter ablation procedures [128], though systemic coagulation markers remained elevated. In Japanese patients receiving rivaroxaban, FM levels decreased after anticoagulation initiation, with both prothrombin time and FM serving as valuable measures of coagulation status [129].

Current evidence suggests that FM reflects the hypercoagulable state in AF and correlates with LAA thrombus formation and cardiovascular risk. While FM elevation indicates prothrombotic conditions, its clinical utility for predicting thromboembolic events appears limited, particularly in anticoagulated patients. Further research is needed to establish optimal FM cut-off values and to determine whether FM measurement can improve risk stratification beyond current clinical scoring systems in specific AF populations.

### 3.3. Ischemic Stroke

Acute ischemic stroke (AIS) is a critical thrombotic event where early diagnosis and stroke subtype classification are essential for optimal therapeutic management. Soluble fibrin, a precursor of stable fibrin polymer formed during active thrombin generation, has proven to be a sensitive marker for detecting hypercoagulable states and arterial thrombosis [121]. FM levels demonstrate significant elevation in AIS patients compared to healthy controls. In a cohort of 162 patients evaluated within six hours of symptom onset, FM levels (23.9 ± 35.2 μg/mL) substantially exceeded those in controls (4.5 ± 1.1 μg/mL), with FM exhibiting superior diagnostic performance to D-dimer [130]. A multicenter analysis of 202 acute cerebral infarction patients confirmed these findings, demonstrating median FM levels of 5.7 μg/mL with a notably low FDP/FM ratio (0.4), suggesting FM’s superiority over traditional markers for detecting arterial thrombosis [121].

Thrombolytic therapy with recombinant tissue plasminogen activator induces dramatic hemostatic changes in acute stroke patients. FM levels show massive increases peaking within 1–3 h post-thrombolysis and persisting up to 72 h, indicating sustained coagulation activation following reperfusion therapy [131].

FM demonstrates particular value in stroke subtype differentiation, especially for identifying cardioembolic sources. In transesophageal echocardiography-guided studies, serum FM levels were markedly elevated in patients with LAA thrombus (88 ± 52 μg/mL) versus those without (14 ± 9 μg/mL), with FM emerging as an independent predictor of thrombus formation [123]. Serial measurements reveal subtype-specific temporal patterns, with FM levels remaining significantly elevated in cardioembolic stroke on days 1–2 compared to non-cardioembolic subtypes [132]. When combined with the National Institute of Health Stroke Scale (NIHSS) score, FM achieves excellent discriminatory capacity for cardiogenic stroke (sensitivity 81.4%, specificity 96.3%) [130].

The prognostic utility of FM extends beyond acute diagnosis. In a prospective cohort of 113 AIS patients followed for approximately one year, elevated FM levels (≥16.5 μg/mL) were associated with substantially higher cerebrovascular event recurrence rates (37.5% vs. 8.6%). Multivariate Cox regression confirmed FM as an independent predictor of stroke recurrence [133].

These studies establish FM as a sensitive biomarker for AIS diagnosis, cardioembolic subtype identification, and recurrence risk stratification. FM measurement provides clinically actionable information for identifying hypercoagulable states and guiding secondary prevention strategies, though larger prospective cohort studies remain necessary for further clinical implementation.

### 3.4. Venous Thromboembolism

Accurate and timely diagnosis of venous thromboembolic complications, including deep vein thrombosis (DVT) and pulmonary embolism (PE), is an essential clinical task [134]. Over the past three decades, numerous studies have evaluated FM as an additional diagnostic marker to confirm or exclude venous thromboembolism (VTE) in various clinical settings.

Early studies established FM as a promising biomarker for VTE diagnosis. Pioneering studies in the 1990s demonstrated that FM measurement could enable early detection of postoperative DVT [4,135]. Subsequently, research expanded to assess FM performance in symptomatic outpatients with suspected VTE. In a study of 426 outpatients with clinically suspected PE, FM testing showed performances comparable to an established D-dimer assay, with both markers demonstrating similar negative predictive values (NPV) for excluding PE [136]. A landmark study of 551 inpatients suspected of having VTE revealed superior diagnostic accuracy for FM compared to D-dimer [137]. Further research confirmed elevated FM levels as an indicator of high thrombotic risk, with significantly higher concentrations observed in VTE patients compared to both non-VTE patients and healthy controls [138]. A comprehensive analysis comparing FM and D-dimer demonstrated that while both markers showed high sensitivity for DVT diagnosis, FM exhibited superior specificity [139]. A study of 119 patients with suspected DVT or PE demonstrated that FM testing achieved 94% sensitivity for PE and 92% sensitivity for DVT [140].

The perioperative period following major orthopedic procedures represents a particularly high-risk setting for VTE development. Extensive research has examined FM’s role in this population. Multiple studies have established FM’s diagnostic value in this population [141,142]. FM demonstrates superior diagnostic accuracy on postoperative day 1 compared to D-dimer, which becomes more reliable from day 4 onward, emphasizing the time-dependent complementary value of both markers [143,144]. A comprehensive study in 326 patients undergoing major orthopedic surgery further validated the utility of fibrin-related markers, demonstrating that while FMC, D-dimer, and fibrinogen and fibrin degradation products (FDP) all showed utility for diagnosing acute VTE, FDP proved less useful for detecting subclinical VTE or predicting postoperative VTE [145]. These temporal differences have enabled development of selective pharmacological prophylaxis strategies based on individual risk assessment using FM combined with other markers such as plasminogen-activator inhibitor-1 (PAI-1) [146,147]. The diagnostic value of FM extends across various orthopedic contexts, including total hip and knee arthroplasty, where FM on postoperative day 1 shows a strong correlation with subsequent DVT development [148,149]. In spine surgery FM measured one day after surgery proved more useful than D-dimer for early VTE diagnosis, achieving 100% sensitivity and 86.3% specificity at a cut-off value of 20.8 μg/mL [150]. Importantly, fondaparinux prophylaxis influences D-dimer levels more significantly than FM levels, suggesting FM may provide more reliable assessment of thrombotic risk in anticoagulated patients [151].

Beyond orthopedic procedures, FM has demonstrated utility in various surgical contexts. Investigation in gastroenterological surgery identified FM as potentially useful for detecting postoperative VTE, though its role in this setting requires further validation [152]. Studies in hepatobiliary-pancreatic surgery demonstrated that early detection of asymptomatic VTE was possible using D-dimer and FM measurements, enabling timely intervention [153]. A comprehensive 2023 analysis confirmed that FM is useful for diagnosing various thrombotic diseases, including VTE [121].

Pregnancy presents unique challenges for VTE diagnosis due to physiological hypercoagulability and D-dimer elevation. Early research identified FM as a potential thrombotic marker during normal pregnancy, with levels remaining relatively stable throughout gestation despite significant D-dimer increases [154]. A large study of 673 women found that FM levels remained normal in 67.2% during late pregnancy and 78.5% during postpartum following vaginal delivery, suggesting utility for VTE screening without changing cut-off values for non-pregnant individuals [155]. Investigation of FM levels throughout pregnancy and postpartum revealed that median FM concentrations showed only slight elevation during pregnancy (6.2 μg/mL) compared to non-pregnant women (4.8 μg/mL), with within-subject biological variation of 20.6% during pregnancy, comparable to non-pregnant women at 16.1% [156]. A comprehensive 2024 study establishing expectancy values for FM in 342 pregnant women across 350 pregnancies demonstrated that FM levels could be calculated irrespective of pregnancy term, unlike other hemostatic markers, which fluctuated significantly [157]. This stability was confirmed in a cohort of 107 pregnant women, where FM levels remained unaffected by gestational age while D-dimer increased progressively throughout pregnancy, and FM proved superior to D-dimer in identifying women at high VTE risk [158]. The stability of FM throughout pregnancy, in contrast to progressive D-dimer elevation, suggests potential advantages for VTE diagnosis in this population [156,157,158]. Utilization of FM for assessing thrombotic risk in women undergoing assisted reproductive technology was recently discussed, with marked elevation of FM associated with hypercoagulation in this patient cohort [159].

Numerous investigations have emphasized the value of combining FM with D-dimer for enhanced diagnostic accuracy, capitalizing on their complementary mechanistic properties. FM offers two fundamental advantages over D-dimer. First, it reflects thrombin activity rather than fibrinolysis, providing independence from fibrinolytic processes that can be influenced by inflammation, malignancy, or physiological conditions such as pregnancy [139,148,160]. Second, FM appears significantly earlier in the thrombotic sequence: levels increase within approximately one day of venous thrombosis onset, compared to 1–2 weeks for D-dimer, enabling earlier detection of hypercoagulable states [148,150]. These complementary characteristics translate into improved clinical performance when both markers are used together. This temporal difference proves particularly valuable in postoperative settings, where FM measurement on day 1 can identify patients developing thrombosis before D-dimer elevation becomes apparent. Similarly, FM’s independence from fibrinolytic activity provides diagnostic advantages when D-dimer specificity is compromised—a common scenario in pregnancy, malignancy, and inflammatory states where fibrinolysis is activated independently of thrombosis [139,140,160]. A comprehensive 2025 analysis examining fibrin-related markers in perioperative VTE confirmed that FM combined with D-dimer provided improved diagnostic performance across various surgical contexts, validating the additive value of this dual-marker approach [161].

The accumulated evidence demonstrates that FM represents a valuable biomarker for VTE diagnosis and risk stratification across diverse clinical settings. While D-dimer remains the established screening tool for VTE, FM offers complementary diagnostic information with potentially superior specificity in certain populations. The marker shows particular promise in two key clinical contexts: first, in orthopedic surgery, where early postoperative measurement enables identification of high-risk patients; and second, in pregnancy, where physiological D-dimer elevation complicates interpretation and FM’s relative stability provides diagnostic advantages. Implementation of FM measurement, particularly in combination with D-dimer and clinical assessment, may enhance diagnostic accuracy and enable more targeted thromboprophylaxis strategies. Future large-scale prospective studies are necessary to establish standardized cut-off values across different clinical contexts, define optimal timing for measurement, and further validate FM’s role in comprehensive VTE diagnostic algorithms.

### 3.5. Disseminated Intravascular Coagulation

Disseminated intravascular coagulation (DIC) is diagnosed based on clinical presentation and specific laboratory findings [162]. The condition is frequently suspected in patients with sepsis, malignancies, or unexplained bleeding and thrombosis. Fibrin monomer has been proposed as a potentially valuable independent predictor of DIC [163]. The utility of FM measurement in DIC has been extensively evaluated using various analytical methods. Early studies employed precipitation-based electrophoretic analysis [164] and high-performance liquid chromatography [165] to detect and quantify FM elevation associated with DIC severity and clinical outcomes. Subsequent studies developed and validated enzyme-linked immunosorbent assay (ELISA) methods [166,167,168]. These investigations consistently demonstrated substantial elevations of plasma soluble fibrin in DIC patients compared to controls, with concentrations reaching 63.4 ± 65.3 μg/mL in DIC patients versus 1.9 ± 1.0 μg/mL in healthy controls [169]. Novel monoclonal antibodies specific for soluble fibrin have been developed, enabling detection without sample pretreatment and facilitating the implementation of automated immunoturbidimetric assays [169].

Recent studies have established reference limits and assessed diagnostic performance using standardized immunoturbidimetric methods. A reference limit of 7.8 μg/mL for FM in healthy individuals has been established, with diagnostic performance evaluated across different DIC stages [170]. FM levels were significantly elevated in both non-overt and overt DIC compared to non-DIC patients, with median values of 5.9 μg/mL in non-DIC, 9.6 μg/mL in non-overt DIC, and 86.6 μg/mL in overt DIC [170]. The diagnostic performance of FM was comparable to D-dimer for both non-overt and overt DIC. However, FM exhibited superior sensitivity, specificity, positive and negative predictive value compared to D-dimer when differentiating overt DIC from non-DIC [170]. These findings were corroborated by additional studies comparing FM with D-dimer across different patient populations [171,172]. A prospective study demonstrated that median FM levels in patients with overt DIC were significantly higher compared to non-overt DIC and non-DIC groups [171]. Importantly, unlike D-dimer, FM levels showed statistically significant differences between non-overt DIC and non-DIC patients. Multivariate analysis identified FM as an independent predictive factor for differentiating overt DIC from non-DIC patients (OR 43.3) and for distinguishing non-overt DIC from non-DIC patients (OR 18.3) [171]. These findings suggest FM may facilitate early diagnosis and prompt therapeutic intervention.

FM demonstrates particular diagnostic value in specific clinical populations where conventional coagulation markers may be unreliable. In patients with liver cirrhosis, FM showed superior diagnostic and prognostic performance compared to D-dimer and FDP, which are confounded by the altered hemostatic profile characteristic of chronic liver disease [173]. Additionally, measurement of FM degradation products—reflecting partial plasmin-mediated degradation—may provide insight into the balance between coagulation and fibrinolysis in DIC patients with accelerated fibrinolytic activity [174].

Beyond diagnosis of established DIC, FM has demonstrated particular value in identifying patients at risk for disease progression. Measuring hemostatic molecular markers before the onset of overt DIC has revealed that FM levels are significantly elevated in the pre-DIC state, with levels being significantly different among DIC, pre-DIC, and non-DIC patients [167,175]. FM was among the most effective markers for pre-DIC diagnosis, exhibiting high sensitivity and specificity [167,175]. The clinical importance of early FM detection has been confirmed through prospective studies. Modified non-overt DIC diagnostic criteria incorporating FM successfully predicted 97.7% of patients who progressed from DIC-negative to DIC-positive status within one week [176]. In a large prospective evaluation of 613 patients with DIC-associated diseases, approximately 8.5% of initially non-DIC patients progressed to overt DIC within one week, with FM emerging as a key discriminating marker [177]. Notably, mortality rates demonstrated a progressive increase from non-DIC (13.7%) to pre-DIC (22.7%) to overt DIC (37.6%), emphasizing that early detection enables timely therapeutic intervention when treatment efficacy is highest [176].

The incorporation of FM into DIC diagnostic and prognostic scoring systems has significantly enhanced their clinical utility. In the context of the International Society of Thrombosis and Haemostasis (ISTH) overt DIC scoring system, using FM instead of D-dimer as the fibrin-related marker substantially enhanced prognostic power, with 28-day mortality reaching 50.0% in FM-positive overt DIC patients compared to 14.0% in those without overt DIC [178]. This superior prognostic performance was confirmed in surgical intensive care cohorts [178]. Subsequent work established specific FM thresholds for the ISTH scoring system: 3.4 μg/mL for moderate increase (2 points) and 138 μg/mL for strong increase (3 points), with disease-specific 3-point cut-offs of 79 μg/mL for infections, 124 μg/mL for solid cancers, and 205 μg/mL for hematopoietic tumors [179]. Markedly elevated FM levels were more predictive of poor outcomes in infectious DIC than in malignancy-associated DIC [179]. Further investigations demonstrated that both DIC score and FM were independent risk factors for mortality [180]. Complementary diagnostic criteria have been developed by the Japanese Society of Thrombosis and Hemostasis, incorporating FM measurement alongside global coagulation tests, reduced platelet count, and antithrombin levels [181]. Evaluation of these criteria in patients with suspected infectious DIC demonstrated high sensitivity and specificity for diagnosing both DIC and pre-DIC states [181].

Substantial evidence shows that fibrin monomer is a valuable biomarker for both diagnosis and management of disseminated intravascular coagulation. FM measurement offers several distinct advantages: it reflects active fibrin formation, representing an early marker of the prothrombotic state; demonstrates comparable or superior diagnostic performance to D-dimer across various DIC stages; and enables identification of patients in the pre-DIC state, facilitating earlier therapeutic intervention [175,176]. Moreover, FM levels correlate with disease severity and clinical outcomes in DIC patients [178,180]. Effective treatment is associated with decreasing FM levels and favorable outcomes, while persistently elevated or rising FM despite intervention indicates poor prognosis [167]. The incorporation of FM into DIC diagnostic algorithms, particularly the ISTH overt DIC scoring system and Japanese diagnostic criteria [176,181], has enhanced both diagnostic accuracy and prognostic assessment. FM demonstrates utility across diverse patient populations, including those with sepsis, malignancies, liver cirrhosis, and obstetric complications, with particularly strong prognostic value in cirrhotic patients [173]. FM measurement provides actionable information for risk stratification in patients with DIC-associated conditions. It complements traditional coagulation parameters by identifying patients at high risk for progression to overt DIC and adverse outcomes. Thus, FM enhances both diagnostic evaluation and prognostic risk assessment in patients with suspected or established DIC.

### 3.6. COVID-19 Associated Coagulopathy

COVID-19-associated coagulopathy represents a complex dysregulated interaction between innate immunity, coagulation, and the vascular endothelium, resulting in a prothrombotic condition with high frequency of thromboembolic complications [182,183]. Recently, it was demonstrated that fibrin plays an essential role in thromboinflammation and neuropathology related to COVID-19 [184]. Despite advances in understanding this coagulopathy, diagnostic criteria and management strategies continue to evolve, particularly regarding hemostatic biomarkers beyond D-dimer [185].

Several studies have investigated the potential utility of FM as a biomarker in COVID-19. In a retrospective study of 164 critically ill patients, FM showed comparable performance to D-dimers in predicting thrombotic events [186]. In 69 critically ill patients with serial measurements, FM levels indicated thrombotic complications but remained normal in most patients without thrombotic events, contrasting with consistently elevated D-dimer [187]. A study of 255 patients with moderate-to-severe COVID-19 found that soluble FM was positive in 20% of cases, correlating with disease severity and prothrombotic parameters [188]. A retrospective analysis of 81 hospitalized patients revealed that elevated FM levels were associated with poor outcomes, with significantly higher levels observed in ICU patients and those with thrombotic complications [189]. A prospective study of 246 patients found that FM during hospitalization predicted in-hospital mortality, though combining FM with D-dimer ratios provided superior predictive value compared to FM alone [190].

However, other studies demonstrated important limitations of FM measurement in COVID-19. In a cohort of 108 patients, only 23% of those with high D-dimers also had increased FM, suggesting that D-dimer levels in COVID-19 might reflect endothelial damage rather than overt DIC [191]. In a Japanese cohort of 247 non-ICU patients, all three documented VTE cases showed elevated FM levels at admission (with D-dimer being normal in one case), although the very small number of VTE events precludes definitive conclusions about comparative diagnostic performance [192]. In a study of 50 patients, FM failed to discriminate between mild and moderate COVID-19 severity and lacked correlation with severity markers, in contrast to D-dimer, which showed clear associations with disease progression [193].

Collectively, these findings suggest that FM’s added value over D-dimer in routine COVID-19 management appears limited, with a lower frequency of elevation and inconsistent predictive value, though it may have a niche role in combination with other markers.

### 3.7. Perspectives and Further Directions

The accumulated evidence demonstrates that fibrin monomer has emerged as a clinically relevant biomarker across diverse thrombotic conditions (Table 1). Three primary applications demonstrate the strongest evidence base and greatest potential for clinical impact: disseminated intravascular coagulation detection, venous thromboembolism diagnosis across diverse populations, and cardioembolic stroke identification.

Fibrin monomer demonstrates considerable utility in DIC diagnosis and risk stratification, representing the application with the strongest clinical evidence. FM provides comparable or superior performance to D-dimer across all DIC stages [170]. Most critically, FM effectively identifies patients in the pre-DIC state before progression to overt coagulopathy [167,175], enabling early therapeutic intervention associated with significantly improved outcomes compared to treatment initiated during established DIC [176,178]. Integration of FM into the ISTH overt DIC score enhanced prognostic power [178]. FM has been successfully incorporated into modified diagnostic criteria by the Japanese Society of Thrombosis and Hemostasis [181], providing a validated framework for implementation. In special populations such as liver cirrhosis patients, FM demonstrates superior prognostic value compared to D-dimer [173], highlighting its utility where conventional markers may be unreliable.

Fibrin monomer shows promise for VTE diagnosis across three distinct clinical contexts, each addressing specific limitations of D-dimer. In pregnancy, FM concentrations remain relatively stable with within-subject biological variation comparable to non-pregnant women [156], contrasting sharply with D-dimer’s progressive elevation throughout gestation. This stability enables VTE diagnosis using standard cut-off values without pregnancy-specific adjustments [155,157], addressing a critical gap in maternal care where VTE represents a leading cause of mortality. In orthopedic surgery, FM measured on postoperative day 1 provides superior diagnostic accuracy compared to D-dimer for early VTE detection, with validated thresholds effectively excluding DVT [143]. Multiple large-scale studies in patients undergoing total hip or knee arthroplasty confirm this advantage [144,145,148], with FM enabling risk-stratified prophylaxis strategies based on individual patient assessment [146,147]. For general VTE diagnosis, FM demonstrates superior diagnostic accuracy compared to D-dimer [137], exhibiting higher specificity while maintaining excellent sensitivity [139]. The complementary nature of FM and D-dimer—reflecting thrombin generation versus fibrinolysis, respectively—suggests that combined assessment provides more comprehensive evaluation than either marker alone [140,160,161].

Finally, in acute ischemic stroke, FM demonstrates particular value for identifying cardioembolic sources. FM levels are markedly elevated in patients with LAA thrombus [123], and when combined with NIHSS score, FM achieves excellent discriminatory capacity for cardiogenic stroke [130]. Beyond acute diagnosis, elevated FM predicts substantially higher cerebrovascular event recurrence [133], enabling identification of high-risk patients requiring intensified secondary prevention strategies.

Despite substantial evidence supporting FM as a valuable biomarker across multiple thrombotic conditions, two fundamental barriers prevent widespread clinical implementation: lack of assay standardization and absence of universal cut-off values.

The most critical limitation is the absence of standardized assay methodologies across laboratories and studies. Various analytical techniques have been employed across studies, including ELISA-based methods [115,166,167,169,178], immunoturbidimetric assays [114,142,159,170,190,194], and latex agglutination tests [138,142,143,154], further complicated by the use of different monoclonal antibodies with varying epitope specificities [6]. A comparative study of two automated FM assays demonstrated that although both reported results in μg/mL, the absolute values obtained differed approximately two-fold (mean 79.43 vs. 32.06 μg/mL) due to different antibodies and manufacturer-specific calibration methods [195]. Critically, there is no international reference material for FM calibration, with each manufacturer using proprietary preparations [195]. The lack of internationally recognized reference materials and standardized calibrators severely limits the comparability of results across studies and institutions. This technical heterogeneity directly contributes to the second major limitation: the absence of universally applicable cut-off values.

The lack of universally applicable FM cut-off values particularly limits its clinical utility. Reference ranges in healthy populations vary considerably across studies, from 0.3 µg/mL to 7.8 µg/mL [140,170], reflecting differences in assay methodology, antibody specificity, and population characteristics [112,154,170]. More critically, optimal diagnostic thresholds demonstrate marked condition-dependent variation: VTE exclusion requires 3.0–4.0 µg/mL for adequate NPV, while DIC diagnosis employs cut-offs ranging from 8.3 µg/mL for mild disease to 205 µg/mL for severe hematopoietic malignancy-associated DIC [136,138,179]. This hundred-fold variation in thresholds makes standardized interpretation nearly impossible. Cut-offs vary not only by clinical purpose (screening versus diagnosis) but also by timing factors such as post-operative day or disease stage. The lack of standardized calibrators and harmonized assay platforms prevents meaningful inter-institutional comparison and hinders the development of evidence-based algorithms. Without consensus reference materials and validated thresholds established through large multicenter trials, FM measurement remains confined to specialized centers using institution-specific criteria [5,6,7].

Beyond these fundamental issues, FM assay adoption is further impeded by limited prospective validation and a lack of clinical decision algorithms. The Japanese Society of Thrombosis and Hemostasis has successfully integrated FM into modified DIC diagnostic criteria [181], but similar initiatives are lacking for other applications. Addressing these limitations through international standardization, establishment of consensus cut-offs, prospective validation studies, and development of evidence-based algorithms represents the essential pathway toward widespread FM implementation. International collaborative efforts, potentially coordinated through organizations such as the ISTH, to develop certified reference materials and harmonized assay protocols would represent a critical step toward resolving these barriers and enabling meaningful cross-study comparisons.

## 4. Conclusions

Fibrin monomer represents a critical intermediate in blood coagulation. It is not only the main structural component of the forming clot but also an active regulator of haemostasis and a valuable clinical biomarker for risk assessment. This review has synthesized the current understanding of FM’s multifaceted roles, spanning molecular mechanisms and clinical applications.

Beyond its classical structural role in clot formation, FM participates in a complex regulatory network of interactions with multiple platelet receptors and plasma components. FM actively modulates coagulation through bidirectional effects on thrombin activity, enhances fibrinolysis via tissue plasminogen activator stimulation and plasmin protection, and triggers platelet activation through GPVI and integrin αIIbβ3 engagement. Recent discoveries concerning its interactions with the GPIb-vWF axis and soluble GPV continue to reveal FM’s sophisticated participation in haemostatic control.

Clinically, FM has emerged as a sensitive biomarker with three well-established applications supported by robust evidence: early detection and risk stratification in disseminated intravascular coagulation, diagnosis of venous thromboembolism in populations where D-dimer shows limitations, and differentiation of cardioembolic from non-cardioembolic stroke. FM generation during the initial phases of coagulation, before D-dimer formation, positions it as an early predictor of upcoming thrombotic events. This enables prognostic assessment for mortality in acute myocardial infarction, stroke recurrence, and progression to overt DIC, potentially facilitating identification of high-risk patients requiring intensified therapeutic strategies.

Currently, broader clinical implementation of FM assays is hindered by limited standardization and absence of universal cut-off values, challenges that future clinical studies should aim to overcome. Nevertheless, as our understanding of FM regulatory mechanisms deepens and analytical methodologies advance, new clinical applications will likely emerge, further expanding FM’s role in the management of thrombotic and haemostatic disorders.

## Figures and Tables

**Table 1:** Summary of fibrin monomer as a clinical biomarker across thrombotic conditions.

Clinical Condition	Key Findings	Diagnostic/ Prognostic Value	Comparison with D-Dimer	References
Myocardial Infarction	Elevated in AMI vs. stable angina; correlates with infarct size and complications	Independent predictor of 3-month mortality; predicts recurrent ischemic events	FM enhances early diagnosis when combined with D-dimer; low FDP/FM ratio distinguishes arterial thrombosis	[111,112,113,114,115,116,117,118,119,120,121]
Atrial Fibrillation	Elevated in patients with LAA thrombus; correlates with abnormal LAA flow	Associated with cardiovascular events and mortality	Performance varies with anticoagulation type	[119,123,124,125,126,127,128,129]
Ischemic Stroke	Markedly elevated; highest elevation in cardioembolic subtype	Predicts LAA thrombus; predicts stroke recurrence	Superior diagnostic performance for cardioembolic stroke	[121,123,130,131,132,133]
VTE	Elevated in DVT/PE; rises earlier than D-dimer	High sensitivity for PE and DVT; enables early postoperative detection	FM superior for early postoperative detection; combined approach improves accuracy	[135,136,137,138,139,140,141,142,143,144,145,146,147,148,149,150,151,152,153,154,155,156,157,158,159,160,161]
VTE in Pregnancy	Elevated FM identifies high VTE risk	Enables VTE diagnosis without pregnancy-specific adjustments	More stable than D-dimer, which increases progressively throughout gestation	[154,155,156,157,158,159]
DIC	Markedly elevated in DIC; significant differences across DIC stages	Identifies pre-DIC state enabling early intervention; independent mortality predictor	Comparable for overt DIC; superior for differentiating non-overt DIC from non-DIC	[163,164,165,166,167,168,169,170,171,172,173,174,175,176,177,178,179,180,181]
COVID-19 Coagulopathy	Elevated in severe cases; correlates with thrombotic complications	Predicts in-hospital mortality when combined with D-dimer	Limited added value over D-dimer alone	[186,187,188,189,190,191,192,193]

Abbreviations: AMI, acute myocardial infarction; DIC, disseminated intravascular coagulation; DVT, deep vein thrombosis; FDP, fibrin degradation products; FM, fibrin monomer; LAA, left atrial appendage; NIHSS, National Institute of Health Stroke Scale; PE, pulmonary embolism; VTE, venous thromboembolism.

## Data Availability

No new data were created or analyzed in this study. Data sharing is not applicable to this article.

## Abbreviations

The following abbreviations are used in this manuscript:

α2-MG	α2-macroglobulin
AF	Atrial fibrillation
ATIII	Antithrombin III
AIS	Acute ischemic stroke
AMI	Acute myocardial infarction
ANAFIE	All Nippon Atrial Fibrillation In the Elderly registry
DIC	Disseminated intravascular coagulation
DVT	Deep vein thrombosis
ELISA	Enzyme-linked immunosorbent assay
FDP	Fibrin/fibrinogen degradation products
FM	Fibrin monomer
FpA	Fibrinopeptide A
FpB	Fibrinopeptide B
FVIII	Factor VIII
FXI	Factor XI
FXIII	Factor XIII
FXIIIa	Activated Factor XIII
GPIb	Glycoprotein Ib
GPV	Glycoprotein V
GPVI	Glycoprotein VI
ICU	Intensive care unit
ISTH	International Society of Thrombosis and Haemostasis
JSTH	Japanese Society of Thrombosis and Hemostasis
LAA	Left atrial appendage
NIHSS	National Institute of Health Stroke Scale
NPV	Negative predictive value
OR	Odds ratio
PE	Pulmonary embolism
SFMC	Soluble fibrin monomer complexes
sGPV	Soluble glycoprotein V
TAFI	Thrombin-activatable fibrinolysis inhibitor
tPA	Tissue-type plasminogen activator
VLDLR	Very low-density lipoprotein receptor
VTE	Venous thromboembolism
vWF	von Willebrand factor
